# Glucarpidase efficacy in mitigating methotrexate toxicity is unaffected by concurrent administration of folinic acid

**DOI:** 10.1007/s00280-026-04880-2

**Published:** 2026-05-08

**Authors:** Martin Wahlestedt, Karin Hansson, Alexandra Bill, Anders Isaksson, Jesper Heldrup, Cornelis Jan Pronk

**Affiliations:** 1 Division of Molecular Hematology, Stem Cell Centre and Wallenberg Center for Molecular Medicine, Lund University, Lund, Sweden; 2 Department of Clinical Chemistry and Pharmacology, Skåne University Hospital, Lund, Sweden; 3 Division of Clinical Chemistry and Pharmacology, Department of Laboratory Medicine, Lund University, Lund, Sweden; 4 Childhood Cancer Center, Skåne University Hospital, Lund, Sweden

**Keywords:** High-dose methotrexate, Cancer, Rescue, Folinic acid, Glucarpidase, Interaction

## Abstract

**Purpose:**

High-dose methotrexate (HDMTX) is a cornerstone in pediatric oncology, but delayed methotrexate elimination (DME) can cause severe toxicity. Rescue therapy typically combines folinic acid (FA) and glucarpidase (GP), yet current guidelines recommend pausing FA around GP administration due to presumed interference. This study aimed to determine whether FA affects GP efficacy in metabolizing methotrexate and to assess the impact on folate metabolites.

**Methods:**

An ex vivo model was developed using human plasma spiked with clinically relevant concentrations of methotrexate and FA, with or without GP. Samples were analyzed by mass spectrometry to quantify methotrexate, FA, and related metabolites. Additional experiments included blood from pediatric patients receiving HDMTX per ALLTogether protocol.

**Results:**

GP rapidly metabolized > 99% of methotrexate across all tested conditions, regardless of FA presence, producing robust DAMPA levels. FA degradation by GP was observed at varying levels, ranging from 8 to 78%. Patient samples confirmed efficient methotrexate clearance by GP, with similar DAMPA production whether FA was present or absent.

**Conclusion:**

Concurrent FA administration does not compromise GP efficacy in neutralizing methotrexate. These findings challenge current recommendations to suspend FA around GP dosing and suggest that continuous FA rescue may be safe and beneficial. Adjustments to FA dosing post-GP may be warranted due to partial FA degradation. Further clinical studies should refine rescue strategies to optimize efficacy and minimize toxicity.

**Supplementary Information:**

The online version contains supplementary material available at 10.1007/s00280-026-04880-2.

## Introduction

**Fig. 1:**
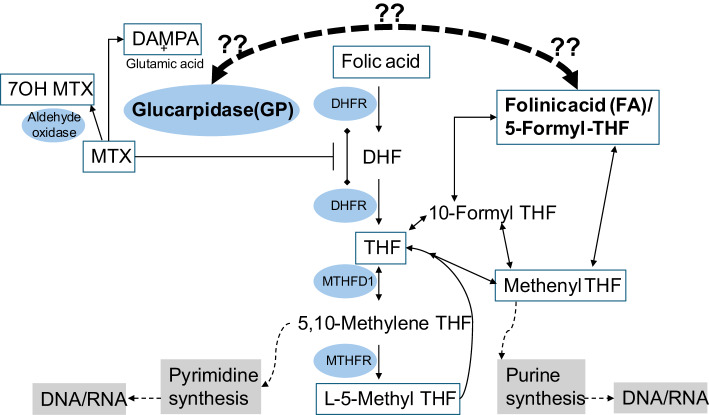
Illustration of folate metabolism depicting major folate isoforms and their converting enzymes, culminating in nucleotide (RNA/DNA) synthesis. Methotrexate (MTX) inhibits dihydrofolate reductase (DHFR), arrests the folate cycle, and thereby suppresses RNA/DNA synthesis; rescue with folinic acid (FA, 5‑formyl‑THF) restores folate cycling and nucleotide synthesis. Question marks indicate potential interfering interactions between Glucarpidase (GP) and FA. Abbreviations: MTX, methotrexate; THF, tetrahydrofolate; DHF, dihydrofolate; DHFR, dihydrofolate reductase; 7OH MTX, 7‑hydroxymethotrexate; DAMPA, 4‑amino‑4‑deoxy‑N10‑methylpteroic acid; MTHFD1, methylenetetrahydrofolate dehydrogenase; MTHFR, methylenetetrahydrofolate reductase. Analytes quantified by the LC‑MS/MS method and presented in Table 1 are in boxes

The folate antagonist aminopterin, the direct precursor to methotrexate (MTX), was the first chemotherapeutic agent employed to induce temporary remission in pediatric leukemia patients in 1948 [1]. Since then, treatment protocols have been refined, and today, high-dose methotrexate (HDMTX) is a cornerstone in the treatment of acute lymphoblastic leukemia (ALL) [2], osteosarcoma, and various brain tumors and lymphomas [3]. Although HDMTX has been extensively utilized for decades, several aspects remain insufficiently explored, including the optimal use of, and interaction between the HDMTX antidote folinic acid (FA, 5-formyl-THF) and glucarpidase (GP) as components of rescue therapy (Fig. 1). MTX and its intracellularly formed polyglutamylated derivatives inhibit the enzyme dihydrofolate reductase (DHFR) to interrupt the folate cycle and deplete the intracellular stores of reduced folates. Polyglutamylation enhances MTX intracellular retention and functions as an intracellular storage vitamin (in the form of reduced folates) following consecutive courses of HDMTX. THF and its derivatives, such as methyl-THF and methylene-THF, are essential for nucleotide and methionine synthesis. High doses of MTX can lead to potentially irreversible toxicities, particularly in cases of delayed MTX elimination (DME). HDMTX treatments necessitate hydration, urine alkalization, and FA rescue to prevent severe toxicities, such as acute kidney injury (AKI), myelotoxicity, and stomatitis [4–6]. Following administration, L-FA is converted to L-5-methyl- tetrahydrofolate (L-5-MTHF), which competes with MTX for cellular entry. Because L-5-MTHF is already in its reduced form, it bypasses the MTX-induced blockage of DHFR and restarts the folat cycle by repleting the reduced folates distal to the block.

**Table 1:** Ex vivo exposure of Folinic Acid does not inhibit Glucarpidase-induced MTX metabolization as measure by DAMPA production

	MTX	7OH MTX	DAMPA	Folic acid	THF	Folinic acid/L-5-Formyl THF (FA)	Methenyl THF	L-5-Methyl THF
Control blood	< 1	< 1	< 1	< 1	< 1	< 1	< 1	19
Control blood + GP	< 1	< 1	< 1	< 1	< 1	< 1	< 1	8
Control blood + FA	< 1	< 1	< 1	7	23	20 850	581	64
Control blood + MTX	46 900	< 1	< 1	2	< 1	1	< 1	22
Control blood + MTX+FA	52 750	< 1	< 1	11	32	24 750	1187	59
Control blood + MTX+GP	316	< 1	42 700	< 1	< 1	< 1	< 1	5
Control blood + FA+GP	< 1	< 1	< 1	< 1	3,5	5120	127	14
Control blood + MTX+FA + GP	299	< 1	43 600	< 1	6	6790	249	12
Patient t24h; - FA, - GP	36 600	18 000	< 1	4	9	< 1	< 1	30
Patient t24h; + FA, - GP	39 000	20 800	< 1	776	1440	1 800 000	178 000	99
Patient t24h; - FA, + GP	209	8960	24 500	8	44	< 1	< 1	< 1
Patient t24h; + FA, + GP	180	14 100	30 300	2	789	50 9000	44 000	10

*Upper panel*: In blood from healthy donors, methotrexate (MTX), folinic acid (FA), and glucarpidase (GP; Voraxaze^®^, BTG) were added ex vivo at final concentrations of 30 µM, 10 µM, and 1 U/ml, respectively, and samples were subsequently analyzed for the indicated metabolites. The results shown represent the average of two individual donors. *Lower panel*: In a patient undergoing HD-MTX treatment, samples were collected 24 h after the start of MTX infusion. A dose of FA, adjusted according to MTX concentrations reported from the medical chart, and a standard dose of GP were then added ex vivo as indicated. Samples were subsequently analyzed by mass spectrometry for the indicated metabolites. Values are presented as concentrations in nmol/L

Notably, studies such as that of Evans et al. [7] focused on defining MTX concentrations required for therapeutic efficacy, whereas FA rescue dosing has continued to be employed largely on empirical grounds [8]. It is conceivable that there exists a delicate balance between the therapeutic effect and toxicity of HDMTX treatment. Insufficient FA administration relative to MTX concentrations results in increased toxicity, whereas excessive FA has been suggested to diminish MTX’s antitumor efficacy (over-rescue) [9–11]. The therapeutic efficacy of HDMTX may be compromised by excessive, too frequent, or too early administration of FA.

The lack of studies and uniformity in FA rescue treatment is likely due to the challenges associated with measuring the interacting metabolites in the folate cycle. We established a method utilizing mass spectrometry (MS) that enables the simultaneous detection of MTX, L-FA, and their relevant metabolites [12]. In certain instances, despite the implementation of supportive measures alongside FA treatment, patients may still experience significant and life-threatening DME, necessitating prompt intervention. In such scenarios, the recombinant bacterial enzyme glucarpidase (GP) could be considered. GP facilitates the elimination of MTX from circulation by cleaving it into DAMPA (4-deoxy-4-amino-N10-methylpteroic acid) and the amino acid glutamate [13]. In addition, GP eliminates circulating L-5-MTHF in the blood, which is the primary biologically active form of folate circulating in the body. However, importantly, GP has no effect on MTX in the tissue compartment or intracellularly. According to the FDA and EMA guidelines for the use of GP (Voraxaze^®^), as well as international expert opinions [13, 14], the administration of FA is not recommended within 2 h before and after GP administration. These recommendations are supported by findings cited in the FDA label, which report that administering GP 2 h before FA rescue reduces plasma concentrations of FA and L-5-MTHF (www.accessdata.fda.gov/drugsatfda_docs/label/2012/125327lbl.pdf).

As GP lack activity outside the bloodstream, this rescue delay with FA may lead to unnecessary prolonged toxicity and rescue time if FA rescue is paused during this period. Concurrently, there is a paucity of research examining the impact of GP on various metabolites within the folic acid cycle, both with and without concurrent FA administration (Fig. 1). In response to this limited understanding, we setup an in vitro system modelling HDMTX to study the interaction between GP and FA at clinically relevant concentrations.

## Materials and methods

For the enzyme assays, 1 ml of Tris-HCl buffer (100 mM, pH 7.3) supplemented with 0.2 mM ZnCl₂ was dispensed into 1.5-ml tubes. Zinc ions were included to ensure proper enzyme activity. Next, different combinations of methotrexate, L- folinic acid and/or Voraxaze (BTG) were added to final concentrations of 30 µM, 10 µM and 1 U/ml respectively. Next, samples were incubated at 37 °C for 5 min before the reaction volumes were transferred to 1.5 ml tubes containing DL-dithiothreitol and ascorbic acid added for final concentrations of 2 mM and 1% respectively.

For experiments using healthy donor blood, samples were collected into Li-heparin–coated vials (Becton Dickinson). Next, 1 ml blood/reaction was transferred into 1.5 ml tubes and 600, 300, 150 and 75 µM methotrexate was added to the indicated tubes. This was followed by light mixing by vortexing and incubation at 37 °C for 30 min. Thereafter, di-sodium-levofolinate (Medac, GmbH) was added to the reactions based on the ALLTogether protocol according to the following formula assuming a body weight of 30 kg: amount of levofolinate in mg: (µM MTX /2) x body weight. Samples were next mixed by gentle vortexing and incubated at 37 °C for 30 min. Finally, Voraxaze (BTG) was added to the indicated samples at a concentration of 1 U/ml, corresponding to the clinical dose for a 30-kg patient, and the samples were incubated at 37 °C for 5 min. Next, the supernatants were acquired by centrifugating the samples and transferred to 1.5 ml tubes containing DL-dithiothreitol and ascorbic acid added for final concentrations of 2 mM and 1% respectively.

Patients received HDMTX with 5000 mg/m^2^ MTX over 24 h. Samples were obtained at 24 h, 42 h and 48 h were treated the same as healthy blood samples. All samples were stored at -80 degrees C for < 2 weeks prior to MS analysis according to Hansson et al. (2021). Experiments were performed with approval from the Swedish Ethical Review Authority.

## Results

We established an ex vivo system designed to simulate HDMTX treatment, incorporating both FA rescue and GP treatment. This system enabled us to investigate the potential interactions between GP and L-FA activity. To validate the feasibility of our approach, we conducted experiments using various combinations of MTX, L-FA, and GP in buffer solution, followed by the measurement of folic acid metabolites via MS [12]. Our findings demonstrated that a brief, 5-minute incubation with GP effectively neutralized MTX and resulted in robust DAMPA production, whereas no DAMPA was detected in control samples lacking GP (Supplementary Fig. 1).

**Fig. 2:**
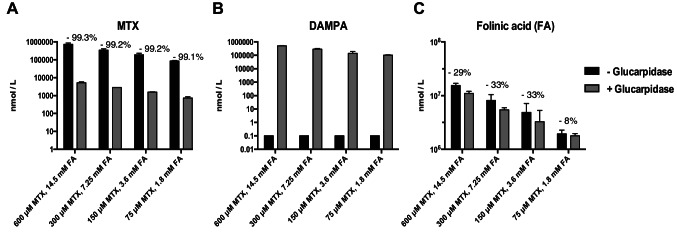
Mass Spectometry analysis for MTX, DAMPA and FA levels in blood samples, in the presence of absence of Glucarpidase. In blood from heatlhy donor, methotrexate and corresponding doses of FA (Folinic acid, or L-5-Formyl THF) were added ex vivo at indicated concentration, in the precense (grey bars) or absence (black bars) of GP. After 5 min of incubation, samples were analysed mass spectometry. Displayed are levels of (**A**) methotrexate, (**B**) DAMPA and (**C**) folinic acid (FA). For FA, reductions in concentrations are shown. Concentrations are on nmol/L. Error bars represent standard deviation

After validating our approach in a controlled ex vivo system, we next simulated severe DME with L-FA rescue and/or GP treatment. We collected blood samples from healthy volunteers and incrementally introduced varying concentrations of MTX and L-FA within a range where GP treatment would be applicable (MTX concentrations 75–600 µM and L-FA concentrations 1.8–14.5 mM, respectively). Subsequently, GP was added to half of the samples, and the plasma supernatants were subjected to MS analysis (Fig. 2). Across all MTX concentrations and corresponding L-FA levels, GP successfully metabolized over 99% of the added MTX, despite the presence of L-FA (Fig. 2A). This was accompanied by a significant production of DAMPA (Fig. 2B). These results were corroborated by a separate experiment, which did not observe any inhibition of GP activity, measuring 42 700 nmol/L and 43 600 nmol/L DAMPA following incubation of MTX-containing blood with GP alone, or with FA + GP, respectively (Table 1, upper part). Next, we evaluated the extent to which GP metabolizes L-FA in addition to MTX. Notably, at MTX concentrations between 150 and 600 µM, approximately 30% of the added L-FA was degraded by GP during a 5-minute incubation, in contrast to only 8% degradation at 75 µM MTX (Fig. 2C). In the presence of lower doses of MTX, the addition of GP resulted in ~ 73% (Table 1, upper part) to ~ 78% (Table 1, lower part) L-FA degradation, exceeding the ~ 30% observed at higher MTX concentrations (150–600 µM, Fig. 2).

Finally, we implemented our experimental design using primary blood samples from pediatric patients receiving HDMTX therapy, administered as a 24-hour infusion of 5000 mg/m², according to the ALLTogether study guidelines (ClinicalTrials.gov ID NCT04307576). Specifically, we collected blood samples at 24, 42, and 48 h following the initiation of treatment. These time-points correspond to routine clinical measurements of MTX concentrations, with L-FA rescue beginning 42 h after treatment initiation in accordance with ALLTogether guidelines. Additionally, GP therapy is typically recommended if MTX concentrations exceed 250 µM at 24 h, 30 µM at 36 h, or 10 µM at 48 h. Ex vivo treatment of T24-, T42-, and T48-hour samples with GP resulted in efficient MTX metabolism in HDMTX samples, as shown by decreased MTX and increased DAMPA levels in MS analysis. Finally, a 24-hour sample was either supplemented with L-FA or left unaltered, followed by GP treatment. In agreement with previous results, the addition of L-FA did not affect GP activity, as similar decreases in MTX (to 209 nmol/L without L-FA and 180 nmol/L with L-FA) and similar increases in DAMPA (to 24 500 nmol/L without L-FA and 30 300 nmol/L with FA) were observed.

## Discussion

Collectively, our study is the first to show that L-FA exposure to GP does not significantly impact GP activity. These findings were derived from an ex vivo setting at clinically relevant MTX/FA concentrations and in samples obtained from patients undergoing HDMTX. The results imply that it may not be necessary to suspend L-FA-rescue therapy during GP administration. This conclusion is particularly noteworthy given that GP exclusively neutralizes MTX in the bloodstream, while L-FA-rescue remains crucial to mitigate the intracellular disruption of the folate cycle caused by MTX. Our findings challenge the current FDA/EMA guidelines and international expert opinion [13, 14], which recommend that FA should not be administered within two hours before or after GP treatment. Contrary to these guidelines, our data indicate that the timing of L-FA administration does not need to be adjusted based on the preceding GP dose. Furthermore, we observed a GP-induced decrease in the concentration of L-FA and its metabolites Methenyl-THF and L-5-Methyl-THF. Clinically, GP affects only blood circulated L-FA and its metabolites, while all L-5-Methyl-THF that has already been transported to the intracellular compartment for rescue activity remains unaffected. After GP, the remaining L-FA in the blood diffuses into the tissue compartment, where it is converted to L-5-MTHF to sustain the rescue process. Therefore, GP administration does not inhibit the intracellular rescue from L-FA treatment. However, given that GP can circulate for up to 48 h, our data suggest considering higher subsequent L-FA doses post-GP and strongly argue against reducing L-FA at any post-GP time point. Importantly, post‑GP L‑FA dosing should not rely on pre-GP MTX measurements, as the majority of the MTX will have been eliminated. Using such values risks inappropriate L‑FA overdosing and may subsequently contribute to electrolyte imbalances. Instead, L-FA dosing should be based a post-GP measurements only, preferably by immunoassays that capture both MTX and DAMPA. Additional studies are needed to optimize the balance between L-FA and MTX for effective rescue. Finally, given the rapid and near-complete neutralization of intravascular MTX by GP, future work should also determine the optimal GP dose. Such studies should aim to maximize therapeutic efficacy and cost-effectiveness while avoiding the risk of PG underdosing.

## Supplementary Information

Below is the link to the electronic supplementary material.

Supplementary Material 1

Supplementary Material 2

## Data Availability

Data is made available upon request.
