# Supplementary material for: Glucarpidase efficacy in mitigating methotrexate toxicity is unaffected by concurrent administration of folinic acid
Source: Cancer Chemother Pharmacol. 2026 May 8;96(1):45. doi: 10.1007/s00280-026-04880-2 (PMC13156163; doi:10.1007/s00280-026-04880-2)
Supplement: Supplementary file 1 — Supplementary Material 1 [file 280_2026_4880_MOESM1_ESM.pdf]

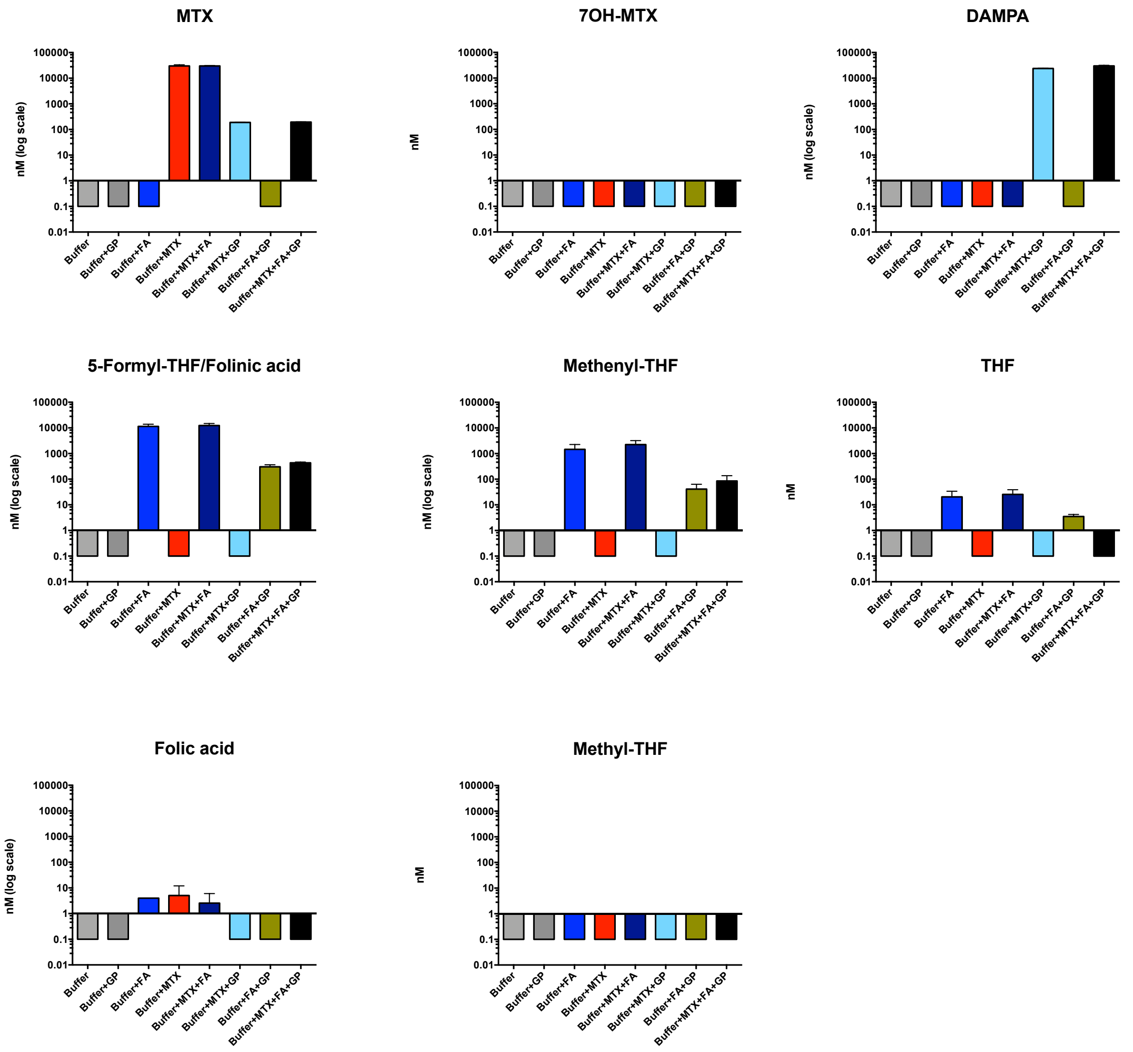

Supplemental Figure 1: Mass Spectometry analysis for MTX, DAMPA and FA levels in buffer samples, in the presence of absence of Glucarpidase.

In buffer samples, Methotrexate, FA and GP were addedd ex vivo as indicated and subsequently samples were analysed for the indicated substrates. Result are average from 2 experiments. MTX, FA and GP (Voraxaze® (BTG)) were added to final concentrations of 30 μM, 10 μM and 1 U/ml respectively. Error bars represent standard deviation
