# Supplementary material for: Glucarpidase efficacy in mitigating methotrexate toxicity is unaffected by concurrent administration of folinic acid
Source: Cancer Chemother Pharmacol. 2026 May 8;96(1):45. doi: 10.1007/s00280-026-04880-2 (PMC13156163; doi:10.1007/s00280-026-04880-2)
Supplement: Supplementary file 2 — Supplementary Material 2 [file 280_2026_4880_MOESM2_ESM.pdf]

Suppl Table 1: Detection of MTX metabolites after ex vivo treatment with Glucarpidase

	MTX	7OH MTX	DAMPA	Folic acid	THF	Folinic acid/L-5-Formyl THF (FA)	Methenyl THF	L-5-Methyl THF
t24h; Nr1, no GP	36600	18000	<1	4	9	<1	<1	30
t24h; Nr2, no GP	17600	13200	<1	20	<1	<1	<1	5
t24h; Nr1, with GP	209	8960	24500	8	44	<1	<1	<1
t24h; Nr2, with GP	296	12600	18700	<1	<1	<1	<1	3
t42h; Nr1, no GP	464	4080	21	<1	7	<1	<1	11
t42h; Nr2, no GP	223	1580	<1	<1	<1	<1	<1	3
t42h; Nr1, with GP	9	3100	527	<1	8	<1	<1	2
t42h; Nr2, with GP	15,9	1210	275	<1	<1	<1	<1	3
t48h; Nr1, no GP	224	2530	9	1	<1	<1	1	157
t48h; Nr2, no GP	181	1270	<1	7	<1	<1	<1	84
t48h; Nr1, with GP	7	1970	278	<1	3	<1	<1	3
t48h; Nr2, with GP	11,3	873	223	<1	<1	<1	<1	4

Two individual ALL patients undergoing HD-MTX treatment; samples were collected at the indicated time points after the start of MTX infusion. GP was then added ex vivo as indicated, and the samples were subsequently analyzed by mass spectrometry for the specified metabolites. Values are presented as concentrations in nmol/L.
